# Supplementary material for: 5-HTT Deficiency in Male Mice Affects Healing and Behavior after Myocardial Infarction
Source: J Clin Med. 2021 Jul 14;10(14):3104. doi: 10.3390/jcm10143104 (PMC8308004; doi:10.3390/jcm10143104)
Supplement: Supplementary file 1 [file jcm-10-03104-s001.zip › jcm-1266884-supplementary.pdf]

SUPPLEMENTAL/ONLINE MATERIALS

5-HTT deficiency in male mice affects healing and behavior after myocardial infarction

Sandy Popp, Angelika Schmitt-Böhrer, Simon Langer, Ulrich Hofmann, Leif Hommers, Kai Schuh, Stefan Frantz, Klaus-Peter Lesch, Anna Frey

Contents

Supplementary Methods Online

Cardiological and immunological methods page 3

Exploration-based approach-avoidance conflict tests page 4

Supplementary Tables Online

Table S1: Echocardiography, body and organ weight data of non-operated mice at 2-3 vs. 4-8 months of age page 5

Table S2: Hemodynamic measurements obtained from non-operated mice at 7 months of age page 6

Table S3: Analysis of gene expression levels in myocardial tissue of non-operated mice by quantitative real-time PCR page 7

Table S4: Quantification of neurotransmitter concentrations in non-operated mouse hearts by HPLC page 8

Table S5: Echocardiography (day 1) and sucrose preference (week 1) of non-surviving MI mice page 10

Table S6: Quantification of infarct size, body, organ weights and collagen content 8 weeks after surgery page 11

Table S7: Statistical analysis of echocardiographic and behavioral measurements obtained from the long-term experiment according to three-way mixed ANOVA page 12

Table S8: Electrocardiographic recordings before and 3 days post-MI page 14

Table S9: Gene expression analysis in myocardial tissue 3 days post-MI page 15

Table S10: Quantification of neurotransmitter concentrations in myocardial tissue 3 days post-MI page 16

Supplementary Figures and Figure Legend Online

Figure S1: Echocardiographic measurements of sham, surviving MI mice with small and large infarction size and non-surviving MI mice at day 1 after surgery page 17

Figure S2: Assessment of body weight change, liquid intake and sucrose preference in sham, surviving MI mice with small and large infarct size and non-surviving MI mice during the first postoperative week page 18

Supplementary Methods Online

Cardiological and Immunological Methods

Echocardiographic Analysis

Serial ultrasound analyses were performed on the Visual Sonics Vevo 1100 mouse echo machine with the echo transducer MS400 (Fujifilm Visualsonics, Toronto, Ontario, Canada) under light isoflurane (about 1.5 vol.%) anesthesia allowing spontaneous respiration. The examinations were performed by a single researcher experienced in rodent echocardiography blinded to the type of operation and treatment. Endocardial borders were traced from two-dimensional short axis images at end-systole and end-diastole utilizing a prototype off-line analysis system (Vevo Lab Workstation Software). Measurements were performed at the mid-papillary muscle level. The end-systolic (smallest) and end-diastolic (largest) cavity areas were determined. Fractional area change (FAC) was calculated using the end-systolic area (ESA) and end-diastolic (EDA) area: (EDA-ESA)/EDA*100. End-systolic diameter (ESD) and end-diastolic diameter (EDD) were measured from two-dimensionally targeted M-mode tracings. Fractional shortening (FS) was calculated: (EDD-ESD)/EDD*100. Only mice with a heart rate greater than 450/min were included in the analysis.

Infarct Size Measurement in the Short-Term Study after Myocardial Infarction (Evans blue/TTC-staining)

Infarct size measurement 3 days after MI via Evans blue/TTC-staining was performed as recently described. Briefly, animals were put under general anesthesia with isoflurane and intubated using a ventilator. The chest was opened and 5% Evans blue (Sigma-Aldrich, Munich, Germany) in PBS was injected into the apex of the heart. After the animals were sacrificed by intra-cardiac injection of saturated KCl solution in PBS, the heart was removed, washed with 0.9% NaCl and frozen in Tissue Tek (Sakura, Alphen aan den Rijn, Netherlands) at −20°C for 30 min. The frozen heart was cut into five parallel transverse slices, which were stained with 2% 2,3,5-Triphenyl-tetrazolium chloride (TTC) (Sigma-Aldrich, Munich, Germany) for 10 min at 37°C. After TTC staining viable myocardium stains blue, boarder-zone stains red and the infarcted areas appear pale. Slices were weighed, imaged and the area of infarction for each section was determined by computerized planimetry using an image analysis software program (VGA Planimetrie 2010 for Canon EOS5D and Planimetrie Report for Microsoft Excel 2010).

Histological Evaluation of the Neutrophil Influx into the Scar

Cryosections of mouse myocardium were prepared in the standard manner. Cells were labelled by the sequential application of the primary rat anti mouse neutrophil antibody (Clone 7/4; Cat-No. LCU8993, Linaris, Wertheim, Germany) or a non-immune immunoglobulin (as a control) followed by labeling with the secondary biotinylated antibody (Vector BA 4001, Rabbit Anti-Rat) and a Vectastatin avidin-biotin complex as indicated by the manufacturer (Vectastain ABC Kit, Vector Laboratories, Burlingame, CA). The slides were washed, incubated with DAB peroxidase substrate solution (prepared by using the DAB substrate Kit from abcam, ab64238) dehydrated, and mounted for light microscopy. The evaluation was performed semi quantitatively by visual classification of neutrophil existence in eight light microscopic 400-fold view fields: "0" indicates absence of high-density positive cells and "3" indicates strongest prevalence of high-density positive cells. Six animals per genotype were analyzed.

FACS analysis of the infarcted tissue

Cell suspensions from the infarct zone from individual hearts were prepared by digestion with collagenase type 2 and protease type XIV (Sigma Aldrich, Germany) and stained according to protocols. A single cell suspension was prepared by filtering the tissue through a 40 µm cell strainer (BD Falcon, Heidelberg, Germany). About 10^6 cells per staining were washed with 100 µl FACS buffer (0.1% BSA, 0.02% NaN3 in PBS) once. To block unspecific binding to Fcγ RIII/II receptors, cells were incubated with saturating amounts of cell culture supernatant of the clone 2.4G2 for 15 min (4°C). Surface antigens were stained for 15 min and cells were washed with FACS buffer one time. The monoclonal antibody anti-mouse CD45 eFluor450 (eBioscience, Frankfurt, Germany) was used for the quantification of leukocytes; to further differentiate between neutrophils (CD45+CD11b+Ly6G+ cells) and monocytes/ macrophages (CD45+CD11b-Ly6G- cells) PE-labelled rat anti-mouse CD11b and Alexa Fluor 647 anti-mouse Ly6G (BD Pharmingen) were used. Cells were analysed in FACS buffer on a BD FACSCanto II flow cytometer (BD, Heidelberg, Germany). Data were analyzed with FlowJo (TreeStar Inc) software (version 7.6.5).

Exploration-based approach-avoidance conflict tests

Elevated Plus Maze

The EPM was made from black Perspex (TSE Systems, Bad Homburg, Germany), semipermeable for infrared light, and illuminated by infrared LEDs from below [26]. The apparatus was elevated to a height of 60 cm above floor level and comprised two opposing open arms (30 x 5 x 0.25 cm, 50 lx) and two opposing closed arms (30 x 5 x 15 cm, 5 lx) extending from a central platform (5 x 5 cm, 30 lx). Mice were placed in the center, facing an open arm, and allowed to freely explore for 10 min. Parameters considered were latency to enter, number of entries, distance traveled, and time spent on the open and closed arms, respectively. An increase in open arm exploration reflects anxiolytic-like behavior, while prolonged closed arm exploration indicates anxiogenic-like behavior. Time spent on the central square was taken as an indicator of risk assessment behavior.

Open Field Test

The OFT consisted of a black Perspex box (50 x 50 x 40 cm, semipermeable for infrared light, TSE Systems, Bad Homburg, Germany) that was illuminated by infrared LEDs from below [26]. The arena was divided into a 30 x 30 cm center zone (120 lx) and the surrounding periphery (50 lx). Mice were placed in a corner and allowed to freely explore for 30 min. Parameters considered were latency to enter, number of entries, distance traveled, and time spent in the center vs. the periphery. Prolonged center exploration reflects anxiolytic-like behavior, while increased exploration of the peripheral zone (thigmotaxis) indicates anxiogenic-like behavior.

Dark-Light Box

The DLB comprised a transparent, brightly lit chamber (40 x 40 x 27 cm, 120 lx) and a small enclosed dark chamber (40 x 20 x 27 cm, 5-10 lx) with a central gate (5 x 5 cm) at floor level to allow access to both compartments [27]. Mice were placed into the dark compartment and allowed to freely explore for 10 min. The latency to head-poke and the number of head-pokes into the lit compartment were taken as indicators of exploratory activity and risk-assessment behavior. The latency to fully enter (with all four paws), number of entries, distance traveled, and time spent in the lit compartment were taken as indicators of risk-taking behavior.

Social Interaction Test

Approach-avoidance behavior towards an unfamiliar conspecific was assessed in the same arena previously used for the OFT (50 x 50 x 40 cm black Perspex box, TSE Systems). The social target was an age-matched C57BL/6 male mouse enclosed in a small wire mesh cage (15 x 6 x 6 cm) to ensure that social approach was initiated by the subject mouse and to permit indirect sensory contact while preventing direct physical (e.g. aggressive) interactions [28]. The wire cage with the social target was located at one end of the arena [29]; experimental mice were placed in the opposite side of the chamber and allowed to freely explore for 10 min. The arena was divided into an interaction zone (26 x 12 cm area surrounding the wire cage) and a corner zone (the two opposing corners of the arena, 12 x 12 cm each). The latency to enter, number of entries, distance traveled, and time spent in the interaction and corner zone, respectively, were taken as indicators of social approach-avoidance behavior.

Supplementary Tables Online

Table S1. Echocardiography, body and organ weight data of non-operated mice at 2-3 vs. 4-8 months of age.

	≤ 3 months			≥ 4 months			Two-way ANOVA		
	5-HTT+/+	5-HTT+/-	5-HTT-/-	5-HTT+/+	5-HTT+/-	5-HTT-/-	Age	Genotype	Age x Genotype
**Echocardiography**	$n = 10$	$n = 18$	$n = 13$	$n = 8$	$n = 8$	$n = 8$			
ESD [mm]	2.42 ± 0.09	2.50 ± 0.09	2.25 ± 0.11	2.65 ± 0.17	2.69 ± 0.27	3.20 ± 0.18	$F_{1,59} = 14.8$, $p < 0.001$	$F_{2,59} = 0.88$, $p = 0.421$	$F_{2,59} = 4.44$, $p = 0.016$
EDD [mm]	3.66 ± 0.11	3.59 ± 0.08	3.43 ± 0.13	3.63 ± 0.15	3.70 ± 0.25	4.03 ± 0.17	$F_{1,59} = 3.63$, $p = 0.062$	$F_{2,59} = 0.24$, $p = 0.790$	$F_{2,59} = 2.61$, $p = 0.082$
FS [%]	33.79 ± 1.50	30.51 ± 1.42	34.59 ± 1.29	27.38 ± 2.34	28.61 ± 3.41	20.80 ± 1.48	$F_{1,59} = 22.0$, $p < 0.001$	$F_{2,59} = 1.12$, $p = 0.334$	$F_{2,59} = 5.11$, $p = 0.009$
ESA [mm^2]	4.52 ± 0.30	5.06 ± 0.28	4.07 ± 0.29	5.17 ± 0.61	5.75 ± 0.68	7.29 ± 0.75	$F_{1,59} = 16.3$, $p < 0.001$	$F_{2,59} = 1.61$, $p = 0.208$	$F_{2,59} = 5.11$, $p = 0.009$
EDA [mm^2]	9.38 ± 0.57	9.14 ± 0.44	8.37 ± 0.49	8.79 ± 0.50	9.42 ± 0.81	10.17 ± 0.66	$F_{1,59} = 1.07$, $p = 0.306$	$F_{2,59} = 0.07$, $p = 0.936$	$F_{2,59} = 2.02$, $p = 0.142$
FAC [%]	50.79 ± 3.74	44.58 ± 1.82	51.31 ± 2.00	41.94 ± 4.11	40.17 ± 2.47	29.23 ± 3.03	$F_{1,59} = 25.8$, $p < 0.001$	$F_{2,59} = 2.24$, $p = 0.116$	$F_{2,59} = 5.42$, $p = 0.007$
HR [bpm]	534.2 ± 16.0	535.8 ± 10.3	557.6 ± 11.0	523.8 ± 24.0	509.4 ± 17.2	497.5 ± 16.4	$F_{1,59} = 6.60$, $p = 0.013$	$F_{2,59} = 0.10$, $p = 0.907$	$F_{2,59} = 1.31$, $p = 0.277$
**Body/organ weights**	$n = 12$	$n = 12$	$n = 10$	$n = 8$	$n = 8$	$n = 8$			
BW [g]	29.3 ± 0.75	29.6 ± 0.68	29.5 ± 1.00	32.4 ± 0.91	31.9 ± 1.97	34.01 ± 1.22	$F_{1,52} = 13.8$, $p < 0.001$	$F_{2,52} = 0.52$, $p = 0.598$	$F_{2,52} = 0.52$, $p = 0.600$
LV [mg]	98.4 ± 3.77	98.5 ± 3.05	97.8 ± 2.89	102.4 ± 3.08	100.5 ± 5.13	97.6 ± 2.95	$F_{1,52} = 0.45$, $p = 0.506$	$F_{2,52} = 0.28$, $p = 0.755$	$F_{2,52} = 0.17$, $p = 0.847$
RV [mg]	27.1 ± 1.19	27.0 ± 0.83	26.9 ± 1.21	28.7 ± 2.19	26.4 ± 1.47	25.8 ± 0.78	$F_{1,52} = 0.01$, $p = 0.965$	$F_{2,52} = 0.74$, $p = 0.482$	$F_{2,52} = 0.61$, $p = 0.545$
Lung [mg]	155.3 ± 4.73	158.1 ± 3.01	151.1 ± 2.50	173.7 ± 9.67	159.6 ± 7.53	157.1 ± 8.16	$F_{1,51} = 3.12$, $p = 0.080$	$F_{2,51} = 1.56$, $p = 0.221$	$F_{2,51} = 1.10$, $p = 0.340$
Spleen [mg]	88.6 ± 7.08	77.5 ± 2.87	76.6 ± 4.39	79.9 ± 3.32	75.8 ± 3.36	78.7 ± 4.88	$F_{1,52} = 0.47$, $p = 0.498$	$F_{2,52} = 1.41$, $p = 0.253$	$F_{2,52} = 0.62$, $p = 0.542$
LV/BW [mg/g]	3.35 ± 0.07	3.33 ± 0.08	3.34 ± 0.13	3.16 ± 0.06	3.17 ± 0.08	2.89 ± 0.13	$F_{1,52} = 11.7$, $p = 0.001$	$F_{2,52} = 1.41$, $p = 0.254$	$F_{2,52} = 1.36$, $p = 0.265$
RV/BW [mg/g]	0.92 ± 0.03	0.91 ± 0.03	0.91 ± 0.02	0.88 ± 0.05	0.83 ± 0.04	0.77 ± 0.03	$F_{1,52} = 10.3$, $p = 0.002$	$F_{2,52} = 1.78$, $p = 0.179$	$F_{2,52} = 1.26$, $p = 0.291$
Lung/BW [mg/g]	5.33 ± 0.18	5.31 ± 0.11	5.15 ± 0.13	5.34 ± 0.20	5.08 ± 0.27	4.65 ± 0.27	$F_{1,51} = 2.29$, $p = 0.136$	$F_{2,51} = 2.60$, $p = 0.084$	$F_{2,51} = 0.90$, $p = 0.414$
Spleen/BW [mg/g]	3.01 ± 0.21	2.63 ± 0.10	2.61 ± 0.18	2.48 ± 0.14	2.41 ± 0.10	2.34 ± 0.18	$F_{1,52} = 6.15$, $p = 0.016$	$F_{2,52} = 1.52$, $p = 0.228$	$F_{2,52} = 0.49$, $p = 0.616$

ESD, end-systolic diameter; EDD, end-diastolic diameter; FS, fractional shortening; ESA, end-systolic area; EDA, end-diastolic area; FAC, fractional area change; HR, heart rate; BW, body weight; LV, left ventricle; RV, right ventricle.

Data are shown as mean±SEM.

Table S2: Hemodynamic measurements obtained from non-operated mice at 7 months of age.

	5-HTT+/+	5-HTT+/-	5-HTT-/-	One-way ANOVA	
**LEFT VENTRICULAR**	***n* = 5**	***n* = 6**	***n* = 5**	$F_{2,13}$	p
SW [mmHg/µl]	1850 ± 195	1327 ± 204	1316 ± 187	2.297	0.140
SV [µl]	21.7 ± 2.23	16.5 ± 2.05	16.0 ± 1.81	2.277	0.142
CO [µl/min]	12832 ± 1417	9191 ± 1100	8432 ± 936	3.886	0.048
Vmax [µl]	46.9 ± 4.79	39.7 ± 3.41	41.5 ± 4.47	0.804	0.469
Vmin [µl]	25.2 ± 4.08	23.2 ± 2.24	25.5 ± 4.97	0.120	0.888
Ves [µl]	26.3 ± 4.00	24.5 ± 2.32	26.9 ± 5.00	0.120	0.888
Ved [µl]	44.6 ± 4.85	38.5 ± 3.32	39.2 ± 4.57	0.610	0.558
Pmax [mmHg]	102 ± 4.64	102 ± 5.54	103 ± 3.68	0.027	0.974
Pmin [mmHg]	-3.55 ± 1.84	0.75 ± 1.85	1.27 ± 1.37	2.231	0.147
Pmean [mmHg]	45.7 ± 0.86	45.2 ± 2.22	46.9 ± 3.58	0.129	0.880
Pdev [mmHg]	105 ± 6.24	101 ± 5.18	102 ± 3.53	0.171	0.844
Pes [mmHg]	99.2 ± 4.25	98.8 ± 5.08	101 ± 4.24	0.046	0.955
Ped [mmHg]	4.83 ± 2.34	7.97 ± 1.95	11.0 ± 2.70	1.667	0.227
HR [bpm]	592 ± 27.7	562 ± 23.0	534 ± 39.3	0.879	0.439
EF [%]	49.2 ± 5.24	42.7 ± 3.35	41.2 ± 6.30	0.695	0.517
Ea [mmHg/µl]	4.79 ± 0.49	6.56 ± 1.08	6.65 ± 0.83	1.374	0.288
Pow max [mmHg/s]	20738 ± 3622	11919 ± 1344	21353 ± 3645	3.497	0.061
dP/dt max [mmHg/s]	10798 ± 1532	9561 ± 951	10329 ± 906	0.311	0.738
dP/dt min [mmHg/s]	-10673 ± 1401	-9830 ± 935	-9318 ± 582	0.422	0.664
dV/dt max [µl/s]	1183 ± 251	872 ± 137	691 ± 48.8	2.151	0.156
dV/dt min [µl/s]	-855 ± 65.8	-565 ± 50.5	-648 ± 70.6	5.892	0.015
P at dV/dt max [mmHg]	0.63 ± 1.17	2.48 ± 2.05	12.25 ± 2.64	8.763	0.004
P at dP/dt max [mmHg]	57.8 ± 5.44	55.4 ± 4.50	56.8 ± 2.61	0.075	0.928
V at dP/dt max [µl]	45.6 ± 4.52	38.6 ± 3.46	40.6 ± 4.31	0.773	0.482
V at dP/dt min [µl]	25.7 ± 4.13	23.7 ± 2.29	25.8 ± 5.02	0.107	0.899
PVA [mmHg/µl]	8477 ± 2815	3020 ± 3772	-7015 ± 9350	1.726	0.216
Tau [ms]	5.15 ± 0.56	5.69 ± 0.34	5.87 ± 0.60	0.544	0.593
**AORTA ASCENDENS**	***n* = 5**	***n* = 6**	***n* = 4**	$F_{2,12}$	p
Pressure Max [mmHg]	101.3 ± 7.1	100.4 ± 5.3	101.4 ± 4.2	0.010	0.990
Pressure Min [mmHg]	64.1 ± 4.1	61.8 ± 3.8	60.6 ± 2.4	0.205	0.818
Pressure height [mmHg]	37.2 ± 3.1	38.6 ± 2.1	40.8 ± 3.4	0.368	0.700
Aortic dp/dt Max [mmHg/s]	3722 ± 159	2897 ± 178	2942 ± 577	2.397	0.133
Aortic dp/dt Min [mmHg/s]	−2164 ± 275	−2128 ± 164	−1819 ± 144	0.734	0.500
dp/dt height [mmHg/s]	5886 ± 283	5025 ± 309	4761 ± 702	1.899	0.192

SW, stroke work; SV, stroke volume; CO, cardiac output; Vmax, maximum volume; Vmin, minimum volume; Ves, end-systolic volume; Ved, end-diastolic volume; Pmax, maximum pressure; Pmin, minimum pressure; Pmean, mean pressure; Pdev, developed pressure; Pes, end-systolic pressure; Ped, end-diastolic pressure; HR, heart rate; EF, ejection fraction; Ea, arterial elastance; Pow max, maximum power; dP/dt max, point of maximum pressure increase; dP/dt min, point of maximum pressure decrease; dV/dt max, point of maximum volume increase; dV/dt min, point of maximum pressure decrease; P, pressure; V, volume; PVA, pressure-volume area; PE, potential energy; CE, cardiac efficiency.

Data are shown as mean±SEM.

Table S3: Analysis of gene expression levels in myocardial tissue of non-operated mice by quantitative real-time PCR.

	Applied Biosystems™ RT-PCR Kit	5-HTT+/+		5-HTT+/-		5-HTT-/-		
		n	Mean ± SEM	*N*	Mean ± SEM	*n*	Mean ± SEM	Statistics
TNF-α	Mm00443260_g1	14	1.000 ± 0.210	14	1.462 ± 0.240	12	1.235 ± 0.162	$F_{2,37}$ = 1.26, p = 0.295
TGF-β	Mm00441724_m1	14	1.000 ± 0.218	14	0.925 ± 0.203	12	1.042 ± 0.269	$F_{2,37}$ = 0.07, p = 0.936
MMP-2	Mm00439498_m1	14	1.000 ± 0.073	14	1.010 ± 0.061	12	1.038 ± 0.089	$F_{2,37}$ = 0.07, p = 0.936
COL-1A1		8	1.000 ± 0.074	8	1.000 ± 0.158	8	0.969 ± 0.107	$F_{2,21}$ = 0.02, p = 0.978
COL-3	Mm01254476_m1	8	1.000 ± 0.129	8	1.075 ± 0.157	8	1.271 ± 0.183	$F_{2,21}$ = 0.78, p = 0.470
5-HT2A	Mm00555764_m1	8	1.000 ± 0.114	8	1.111 ± 0.267	8	1.006 ± 0.186	$F_{2,21}$ = 0.10, p = 0.907
5-HT2B	Mm00434123_m1	6	1.000 ± 0.121	6	0.890 ± 0.256	6	0.773 ± 0.230	$F_{2,15}$ = 0.29, p = 0.752
SERT	Mm00439391_m1	6	1.000 ± 0.312	6	1.530 ± 0.594	6	0.000 ± 0.000	$\chi^2_{(2)}$ = 12.0, p = 0.002
COL-1A2	Mm00483888_m1	2	1.000 ± 0.000	2	1.190 ± 0.041	2	1.112 ± 0.077	$\chi^2_{(2)}$ = 3.82, p = 0.148
MMP-3		2	1.000 ± 0.086	2	0.946 ± 0.000	2	1.120 ± 0.285	$\chi^2_{(2)}$ = 0.08, p = 0.963
MMP-13		2	1.000 ± 0.254	2	0.764 ± 0.092	2	0.920 ± 0.064	$\chi^2_{(2)}$ = 1.40, p = 0.497
SPARC		2	1.000 ± 0.017	2	1.216 ± 0.126	2	1.023 ± 0.106	$\chi^2_{(2)}$ = 2.00, p = 0.368
ACTA2		2	1.000 ± 0.121	2	1.046 ± 0.036	2	0.707 ± 0.085	$\chi^2_{(2)}$ = 3.43, p = 0.180

TGF-β, transforming growth factor β; TNF-α, tumor necrosis factor α; IL-6, interleukin 6; IL-10, interleukin 10; MMP-2, matrix metalloproteinase 2; MMP-3, matrix metalloproteinase 3; MMP-13, matrix metalloproteinase 13; Col-1α1, collagen 1α1; Col-1α2, collagen 1α2; Col-3, collagen 3; SPARC, secreted protein acidic and rich in cysteine; ACTA2, alpha smooth muscle actin 2, 5HT2A, serotonin receptor 2A; 5HT2B, serotonin receptor 2B; SERT, serotonin transporter.

Data are shown as mean±SEM.

Applied Biosystems™ RT-PCR Kit for GAPDH as the reference gene: Mm99999915_g1.

Table S4: Quantification of neurotransmitter concentrations in non-operated mouse hearts by HPLC.

	5-HTT+/+ *n* = 4	5-HTT+/- *n* = 3	5-HTT-/- *n* = 4	Welch's ANOVA
**Concentration [ng/g]**				
NE	3952 ± 650	5843 ± 2432	4040 ± 368	$F_{2,3.82} = 0.24$, $p = 0.797$
EPI	180 ± 65	158 ± 44	201 ± 31	$F_{2,4.75} = 0.28$, $p = 0.764$
MHPG	235 ± 75	199 ± 43	289 ± 52	$F_{2,5.28} = 0.79$, $p = 0.499$
5-HTP	685 ± 182	688 ± 157	880 ± 199	$F_{2,5.29} = 0.31$, $p = 0.746$
5-HT	45 ± 9	31 ± 11	42 ± 4	$F_{2,4.01} = 0.46$, $p = 0.659$
5-HIAA	82 ± 19	99 ± 58	68 ± 10	$F_{2,3.79} = 0.28$, $p = 0.773$
DA	31 ± 7	37 ± 6	41 ± 7	$F_{2,5.27} = 0.42$, $p = 0.678$
DOPAC	13 ± 3	21 ± 11	36 ± 9	$F_{2,3.77} = 2.27$, $p = 0.225$
HVA	120 ± 19	127 ± 27	150 ± 14	$F_{2,4.52} = 0.80$, $p = 0.504$
**Turnover rate**				
MHPG/NE	0.058 ± 0.015	0.050 ± 0.020	0.074 ± 0.015	$F_{2,4.81} = 0.48$, $p = 0.646$
MHPG/EPI	1.372 ± 0.098	1.330 ± 0.136	1.428 ± 0.071	$F_{2,4.50} = 0.22$, $p = 0.814$
MHPG/(NE+EPI)	0.055 ± 0.014	0.048 ± 0.019	0.070 ± 0.013	$F_{2,4.79} = 0.50$, $p = 0.636$
5-HIAA/5-HT	2.116 ± 0.698	7.794 ± 6.797	1.671 ± 0.274	$F_{2,3.53} = 0.48$, $p = 0.653$
DOPAC/DA	0.538 ± 0.181	0.597 ± 0.308	0.862 ± 0.244	$F_{2,4.62} = 0.51$, $p = 0.631$
HVA/DA	4.870 ± 1.567	3.842 ± 1.374	4.138 ± 1.067	$F_{2,4.94} = 0.11$, $p = 0.896$
(DOPAC+HVA)/DA	5.409 ± 1.710	4.439 ± 1.277	5.000 ± 1.031	$F_{2,4.96} = 0.10$, $p = 0.906$

NE, norepinephrine; EPI, epinephrine; MHPG, 3-methoxy-4-hydroxyphenylglycol; 5-HTP, 5-hydroxytryptophan; 5-HT, 5-hydroxytryptamine; 5-HIAA, 5-hydroxyindoleacetic acid; DA, dopamine; DOPAC, 3,4-dihydroxyphenylacetic acid; HVA, homovanillic acid.
Data are shown as mean±SEM.

Table S5: Echocardiography (day 1) and sucrose preference (week 1) of non-surviving MI mice.

Echocardiography	5-HTT+/+ *n* = 9–10	5-HTT+/- *n* = 6–7	5-HTT-/- *n* = 11	One-way ANOVA
ESD [mm]	4.45 ± 0.23	4.30 ± 0.27	4.19 ± 0.11	$F_{2,24}$ = 0.54, p = 0.592
EDD [mm]	4.79 ± 0.19	4.77 ± 0.19	4.62 ± 0.13	$F_{2,24}$ = 0.34, p = 0.718
FS [%]	7.38 ± 1.43	10.2 ± 2.59	9.27 ± 0.80	$F_{2,24}$ = 0.90, p = 0.420
ESA [mm^2]	14.4 ± 1.16	14.8 ± 0.89	13.0 ± 0.70	$F_{2,25}$ = 1.05, p = 0.365
EDA [mm^2]	16.5 ± 1.32	17.0 ± 0.83	15.1 ± 0.62	$F_{2,25}$ = 1.09, p = 0.353
FAC [%]	13.2 ± 1.21	13.3 ± 2.60	14.3 ± 2.02	$F_{2,25}$ = 0.11, p = 0.896
HR [bpm]	444 ± 19.4	443 ± 13.7	491 ± 13.0	$F_{2,24}$ = 3.26, p = 0.056
**Sucrose preference test**	***n* = 5**	***n* = 7**	***n* = 6**	
Total liquid intake [ml]	1.23 ± 0.37	1.40 ± 0.38	0.88 ± 0.11	$F_{2,15}$ = 0.74, p = 0.492
Sucrose preference [%]	52.2 ± 7.29	65.2 ± 4.95	54.4 ± 6.27	$F_{2,15}$ = 1.40, p = 0.276

ESD, end-systolic diameter; EDD, end-diastolic diameter; FS, fractional shortening; ESA, end-systolic area; EDA, end-diastolic area; FAC, fractional area change; HR, heart rate.
Data are shown as mean±SEM.

Table S6: Quantification of infarct size, body, organ weights and collagen content 8 weeks after surgery.

	Sham			MI <30%			MI >30%		Two-way ANOVA		
	5-HTT+/+	5-HTT+/-	5-HTT-/-	5-HTT+/+	5-HTT+/-	5-HTT-/-	5-HTT+/+	5-HTT+/-	Group	Genotype	Group x Genotype
**Body/organ weights**	***n* = 9**	***n* = 11**	***n* = 3**	***n* = 8**	***n* = 10**	***n* = 5**	***n* = 7**	***n* = 9**			
Infarct size [%]	N/A	N/A	N/A	11.2 ± 4.02	4.99 ± 1.88	5.24 ± 3.50	59.0 ± 3.88	50.8 ± 3.14	$F_{1,34}$=222, p<0.001	$F_{2,34}$=1.17, p=0.324	$F_{1,34}$=0.11, p=0.739
BW [g]	29.9 ± 1.05	31.1 ± 0.58	32.3 ± 1.31	32.7 ± 1.23	30.3 ± 0.88	32.8 ± 0.75	33.6 ± 1.01	30.8 ± 0.87	$F_{2,54}$=1.76, p=0.182	$F_{2,54}$=1.32, p=0.276	$F_{3,54}$=2.11, p=0.110
LV [mg]	99.6 ± 3.20	99.8 ± 2.74	97.0 ± 4.39	115.2 ± 6.28	108.0 ± 4.08	106.2 ± 3.85	137.3 ± 6.99	137.3 ± 7.89	$F_{2,54}$=27.9, p<0.001	$F_{2,54}$=0.46, p=0.634	$F_{3,54}$=0.24, p=0.866
RV [mg]	23.9 ± 1.15	27.0 ± 1.04	32.7 ± 4.50	29.1 ± 1.25	27.6 ± 1.17	28.5 ± 2.39	38.0 ± 3.93	31.8 ± 2.89	$F_{2,54}$=11.3, p<0.001	$F_{2,54}$=1.27, p=0.290	$F_{3,54}$=2.45, p=0.073
Lung [mg]	151.5 ± 4.71	158.3 ± 3.35	150.4 ± 1.43	159.4 ± 5.02	153.8 ± 5.41	153.9 ± 4.58	169.1 ± 11.7	171.1 ± 14.5	$F_{2,54}$=2.21, p=0.120	$F_{2,54}$=0.08, p=0.921	$F_{3,54}$=0.24, p=0.870
Spleen [mg]	76.7 ± 5.15	83.6 ± 4.32	65.8 ± 6.27	78.8 ± 3.03	81.3 ± 5.14	80.0 ± 6.92	86.8 ± 6.37	79.0 ± 7.17	$F_{2,50}$=0.63, p=0.537	$F_{2,50}$=0.77, p=0.467	$F_{3,50}$=0.63, p=0.598
LV/BW [mg/g]	3.33 ± 0.04	3.21 ± 0.06	3.02 ± 0.24	3.56 ± 0.24	3.58 ± 0.13	3.24 ± 0.08	4.07 ± 0.14	4.45 ± 0.18	$F_{2,54}$=27.0, p<0.001	$F_{2,54}$=1.65, p=0.202	$F_{3,54}$=1.17, p=0.330
RV/BW [mg/g]	0.81 ± 0.04	0.87 ± 0.03	1.03 ± 0.18	0.90 ± 0.05	0.91 ± 0.04	0.87 ± 0.08	1.13 ± 0.13	1.03 ± 0.08	$F_{2,54}$=7.59, p=0.001	$F_{2,54}$=0.73, p=0.489	$F_{3,54}$=1.33, p=0.275
Lung/BW [mg/g]	5.07 ± 0.06	5.11 ± 0.16	4.67 ± 0.16	4.92 ± 0.23	5.09 ± 0.12	4.71 ± 0.19	5.00 ± 0.25	5.56 ± 0.43	$F_{2,54}$=0.77, p=0.467	$F_{2,54}$=1.05, p=0.358	$F_{3,54}$=0.47, p=0.701
Spleen/BW [mg/g]	2.48 ± 0.11	2.70 ± 0.14	2.05 ± 0.24	2.35 ± 0.13	2.68 ± 0.14	2.46 ± 0.24	2.58 ± 0.16	2.59 ± 0.26	$F_{2,49}$=0.10, p=0.905	$F_{2,49}$=2.58, p=0.086	$F_{3,49}$=0.73, p=0.538
**Collagen content**	***n* = 6**	***n* = 8**	***n* = 2**	***n* = 6**	***n* = 6**	***n* = 3**	***n* = 7**	***n* = 9**			
Septum	0.21 ± 0.03	0.34 ± 0.09	0.15 ± 0.02	0.18 ± 0.03	0.38 ± 0.13	1.15 ± 0.62	0.24 ± 0.07	0.76 ± 0.17	$F_{2,39}$=1.64, p=0.207	$F_{2,39}$=2.49, p=0.096	$F_{3,39}$=2.99, p=0.043
Border zone	N/A	N/A	N/A	N/A	N/A	N/A	1.14 ± 0.23	2.67 ± 0.58	N/A	$T_{9.1}$=-2.45, p=0.037	N/A
Infarct scar	N/A	N/A	N/A	N/A	N/A	N/A	77.9 ± 2.85	72.2 ± 4.21	N/A	$T_{14.0}$=1.06, p=0.308	N/A

BW, body weight; LV, left ventricle; RV, right ventricle.

Data are shown as mean±SEM.

Table S7: Statistical analysis of echocardiographic and behavioral measurements obtained from the long-term experiment according to three-way mixed ANOVA.

	Between-Subjects Effects			Within-Subjects Effects			
	Group	Genotype	Group x Genotype	Time	Time x Group	Time x Genotype	Time x Group x Genotype
**Echocardiography**							
ESD	$F_{2,54} = 49.0$, $p < 0.001$	$F_{2,54} < 0.01$, $p = 0.998$	$F_{3,54} = 0.52$, $p = 0.667$	$F_{2,91} = 7.04$, $p = 0.003$	$F_{3,91} = 16.5$, $p < 0.001$	$F_{3,91} = 1.39$, $p = 0.248$	$F_{5,91} = 0.98$, $p = 0.434$
EDD	$F_{2,54} = 37.9$, $p < 0.001$	$F_{2,54} = 0.08$, $p = 0.925$	$F_{3,54} = 0.90$, $p = 0.449$	$F_{2,95} = 8.93$, $p < 0.001$	$F_{4,95} = 17.8$, $p < 0.001$	$F_{4,95} = 1.41$, $p = 0.242$	$F_{5,95} = 1.27$, $p = 0.280$
FS	$F_{2,54} = 55.9$, $p < 0.001$	$F_{2,54} = 0.40$, $p = 0.672$	$F_{3,54} = 0.04$, $p = 0.991$	$F_{2,89} = 3.85$, $p = 0.032$	$F_{3,89} = 1.29$, $p = 0.284$	$F_{3,89} = 1.94$, $p = 0.130$	$F_{5,89} = 0.58$, $p = 0.716$
ESA	$F_{2,54} = 54.7$, $p < 0.001$	$F_{2,54} = 0.09$, $p = 0.913$	$F_{3,54} = 1.28$, $p = 0.289$	$F_{2,91} = 11.2$, $p < 0.001$	$F_{3,91} = 16.2$, $p < 0.001$	$F_{3,91} = 0.81$, $p = 0.504$	$F_{5,91} = 0.38$, $p = 0.861$
EDA	$F_{2,54} = 47.8$, $p < 0.001$	$F_{2,54} = 0.29$, $p = 0.749$	$F_{3,54} = 1.63$, $p = 0.194$	$F_{2,94} = 11.7$, $p < 0.001$	$F_{3,94} = 16.6$, $p < 0.001$	$F_{3,94} = 0.59$, $p = 0.651$	$F_{5,94} = 0.44$, $p = 0.826$
FAC	$F_{2,54} = 53.2$, $p < 0.001$	$F_{2,54} = 1.08$, $p = 0.345$	$F_{3,54} = 0.12$, $p = 0.947$	$F_{2,88} = 2.01$, $p = 0.148$	$F_{3,88} = 1.43$, $p = 0.239$	$F_{3,88} = 2.81$, $p = 0.040$	$F_{5,88} = 0.73$, $p = 0.598$
HR	$F_{2,54} = 6.60$, $p = 0.003$	$F_{2,54} = 0.63$, $p = 0.537$	$F_{3,54} = 1.35$, $p = 0.269$	$F_{2,108} = 29.5$, $p < 0.001$	$F_{4,108} = 2.52$, $p = 0.046$	$F_{4,108} = 0.43$, $p = 0.791$	$F_{6,108} = 0.53$, $p = 0.786$
**Sucrose Preference**							
Body weight change	$F_{2,54} = 0.88$, $p = 0.423$	$F_{2,54} = 4.80$, $p = 0.012$	$F_{3,54} = 1.70$, $p = 0.177$	$F_{2,100} = 370$, $p < 0.001$	$F_{4,100} = 8.16$, $p < 0.001$	$F_{4,100} = 2.93$, $p = 0.027$	$F_{6,100} = 1.98$, $p = 0.081$
Total fluid intake	$F_{2,54} = 0.11$, $p = 0.898$	$F_{2,54} = 9.54$, $p < 0.001$	$F_{3,54} = 0.11$, $p = 0.955$	$F_{2,133} = 5.87$, $p = 0.002$	$F_{5,133} = 8.38$, $p < 0.001$	$F_{5,133} = 0.36$, $p = 0.873$	$F_{7,133} = 0.56$, $p = 0.800$
Sucrose preference	$F_{2,54} = 0.46$, $p = 0.635$	$F_{2,54} = 0.11$, $p = 0.894$	$F_{3,54} = 0.49$, $p = 0.692$	$F_{4,200} = 2.35$, $p = 0.060$	$F_{7,200} = 1.21$, $p = 0.298$	$F_{7,200} = 0.72$, $p = 0.667$	$F_{11,200} = 0.53$, $p = 0.885$
**Elevated Plus Maze**							
Distance traveled	$F_{2,54} = 2.04$, $p = 0.140$	$F_{2,54} = 0.50$, $p = 0.607$	$F_{3,54} = 1.07$, $p = 0.369$	$F_{4,216} = 35.4$, $p < 0.001$	$F_{8,216} = 1.02$, $p = 0.421$	$F_{8,216} = 2.26$, $p = 0.024$	$F_{12,216} = 0.66$, $p = 0.793$
Total arm entries	$F_{2,54} = 0.31$, $p = 0.733$	$F_{2,54} = 1.45$, $p = 0.243$	$F_{3,54} = 0.54$, $p = 0.654$	$F_{4,216} = 2.03$, $p = 0.091$	$F_{8,216} = 1.19$, $p = 0.304$	$F_{8,216} = 1.84$, $p = 0.072$	$F_{12,216} = 1.23$, $p = 0.262$
Open arm entries	$F_{2,54} = 2.69$, $p = 0.077$	$F_{2,54} = 0.36$, $p = 0.700$	$F_{3,54} = 0.85$, $p = 0.475$	$F_{4,216} = 26.9$, $p < 0.001$	$F_{8,216} = 0.62$, $p = 0.761$	$F_{8,216} = 1.28$, $p = 0.256$	$F_{12,216} = 0.45$, $p = 0.939$
Open arm time	$F_{2,54} = 1.15$, $p = 0.325$	$F_{2,54} = 1.09$, $p = 0.344$	$F_{3,54} = 0.98$, $p = 0.408$	$F_{2,132} = 10.7$, $p < 0.001$	$F_{5,132} = 1.05$, $p = 0.392$	$F_{5,132} = 1.04$, $p = 0.399$	$F_{7,132} = 0.42$, $p = 0.897$
Center time	$F_{2,54} = 2.77$, $p = 0.071$	$F_{2,54} = 0.84$, $p = 0.436$	$F_{3,54} = 3.39$, $p = 0.024$	$F_{2,124} = 29.3$, $p < 0.001$	$F_{5,124} = 2.27$, $p = 0.057$	$F_{5,124} = 1.43$, $p = 0.222$	$F_{7,124} = 1.29$, $p = 0.263$
Fecal boli	$F_{2,54} = 0.28$, $p = 0.754$	$F_{2,54} = 2.40$, $p = 0.101$	$F_{3,54} = 0.34$, $p = 0.799$	N/A	N/A	N/A	N/A
**Open Field**							
Distance traveled	$F_{2,54} = 0.81$, $p = 0.451$	$F_{2,54} = 12.7$, $p < 0.001$	$F_{3,54} = 0.82$, $p = 0.487$	$F_{3,183} = 18.3$, $p < 0.001$	$F_{7,183} = 2.96$, $p = 0.006$	$F_{7,183} = 1.21$, $p = 0.300$	$F_{10,183} = 1.34$, $p = 0.212$
Center entries	$F_{2,54} = 3.40$, $p = 0.041$	$F_{2,54} = 7.31$, $p = 0.002$	$F_{3,54} = 0.54$, $p = 0.658$	$F_{4,212} = 17.7$, $p < 0.001$	$F_{8,212} = 1.55$, $p = 0.143$	$F_{8,212} = 1.04$, $p = 0.408$	$F_{12,212} = 0.75$, $p = 0.704$
Center time	$F_{2,54} = 4.49$, $p = 0.016$	$F_{2,54} = 4.21$, $p = 0.020$	$F_{3,54} = 0.34$, $p = 0.782$	$F_{3,187} = 14.1$, $p < 0.001$	$F_{7,187} = 1.38$, $p = 0.219$	$F_{7,187} = 1.09$, $p = 0.374$	$F_{10,187} = 0.47$, $p = 0.912$
Center distance	$F_{2,54} = 6.04$, $p = 0.004$	$F_{2,54} = 6.69$, $p = 0.003$	$F_{3,54} = 0.14$, $p = 0.938$	$F_{4,219} = 27.1$, $p < 0.001$	$F_{8,219} = 0.95$, $p = 0.474$	$F_{8,219} = 1.31$, $p = 0.238$	$F_{12,219} = 0.82$, $p = 0.629$
Fecal boli	$F_{2,54} = 7.08$, $p = 0.002$	$F_{2,54} = 3.55$, $p = 0.036$	$F_{3,54} = 0.18$, $p = 0.909$	N/A	N/A	N/A	N/A
**Dark/Light Box**							
Head-pokes into light	$F_{2,54} = 2.31$, $p = 0.109$	$F_{2,54} = 5.54$, $p = 0.007$	$F_{3,54} = 0.34$, $p = 0.794$	$F_{3,180} = 28.4$, $p < 0.001$	$F_{7,180} = 1.17$, $p = 0.325$	$F_{7,180} = 1.42$, $p = 0.203$	$F_{10,180} = 0.77$, $p = 0.605$
Entries into light	$F_{2,54} = 2.84$, $p = 0.067$	$F_{2,54} = 6.38$, $p = 0.003$	$F_{3,54} = 0.31$, $p = 0.822$	$F_{4,190} = 27.7$, $p < 0.001$	$F_{7,190} = 1.19$, $p = 0.310$	$F_{7,190} = 1.29$, $p = 0.257$	$F_{11,190} = 0.51$, $p = 0.887$
Time in light	$F_{2,54} = 0.54$, $p = 0.587$	$F_{2,54} = 8.44$, $p = 0.001$	$F_{3,54} = 2.65$, $p = 0.058$	$F_{3,154} = 26.8$, $p < 0.001$	$F_{6,154} = 0.95$, $p = 0.462$	$F_{6,154} = 1.36$, $p = 0.237$	$F_{9,154} = 0.89$, $p = 0.531$
Distance in light	$F_{2,54} = 1.20$, $p = 0.310$	$F_{2,54} = 9.44$, $p < 0.001$	$F_{3,54} = 1.19$, $p = 0.322$	$F_{3,175} = 24.8$, $p < 0.001$	$F_{6,175} = 0.80$, $p = 0.577$	$F_{6,175} = 1.62$, $p = 0.139$	$F_{10,175} = 0.66$, $p = 0.758$
Fecal boli	$F_{2,54} = 0.60$, $p = 0.550$	$F_{2,54} = 2.48$, $p = 0.093$	$F_{3,54} = 1.02$, $p = 0.392$	N/A	N/A	N/A	N/A
**Social Interaction**							
Distance traveled	$F_{2,53} = 0.26$, $p = 0.770$	$F_{2,53} = 9.35$, $p < 0.001$	$F_{3,53} = 0.03$, $p = 0.994$	$F_{3,176} = 17.9$, $p < 0.001$	$F_{7,176} = 1.62$, $p = 0.136$	$F_{7,176} = 2.42$, $p = 0.024$	$F_{10,176} = 0.83$, $p = 0.602$

Interaction zone entries	$F_{2,53} = 1.61$, $p = 0.210$	$F_{2,53} = 5.69$, $p = 0.006$	$F_{3,53} = 0.44$, $p = 0.725$	$F_{3,166} = 23.8$, $p < 0.001$	$F_{6,166} = 1.55$, $p = 0.162$	$F_{6,166} = 1.98$, $p = 0.068$	$F_{9,166} = 0.81$, $p = 0.613$
Interaction zone time	$F_{2,53} = 1.38$, $p = 0.261$	$F_{2,53} = 5.32$, $p = 0.008$	$F_{3,53} = 0.17$, $p = 0.916$	$F_{2,130} = 31.5$, $p < 0.001$	$F_{5,130} = 0.61$, $p = 0.688$	$F_{5,130} = 2.63$, $p = 0.028$	$F_{7,130} = 0.43$, $p = 0.891$
Corner time	$F_{2,53} = 0.15$, $p = 0.864$	$F_{2,53} = 6.67$, $p = 0.003$	$F_{3,53} = 0.15$, $p = 0.927$	$F_{2,115} = 14.3$, $p < 0.001$	$F_{4,115} = 1.16$, $p = 0.333$	$F_{4,115} = 3.58$, $p = 0.007$	$F_{7,115} = 0.70$, $p = 0.663$
Fecal boli	$F_{2,53} = 1.03$, $p = 0.364$	$F_{2,53} = 0.12$, $p = 0.888$	$F_{3,53} = 0.62$, $p = 0.603$	N/A	N/A	N/A	N/A

ESD, end-systolic diameter; EDD, end-diastolic diameter; FS, fractional shortening; ESA, end-systolic area; EDA, end-diastolic area; FAC, fractional area change; HR, heart rate; N/A, not applicable.

Table S8: Electrocardiographic recordings before and 3 days post-MI.

	Baseline				3 days post-MI			
	5-HTT+/+	5-HTT+/-	5-HTT-/-	Welch's ANOVA	5-HTT+/+	5-HTT+/-	5-HTT-/-	Welch's ANOVA
	$n = 8$	$n = 14$	$n = 9$		$n = 6$	$n = 9$	$n = 4$	
Arrhythmia	1.50 ± 0.63	0.93 ± 0.29	0.78 ± 0.57	$F_{2,13.45} = 0.40$, $p = 0.676$	0.17 ± 0.17	0.56 ± 0.56	0.75 ± 0.75	$F_{2,6.44} = 0.44$, $p = 0.663$
Heart rate [bpm]	460.9 ± 15.4	483.0 ± 13.9	501.8 ± 13.0	$F_{2,17.13} = 1.99$, $p = 0.167$	602.7 ± 31.2	560.0 ± 16.2	545.8 ± 29.5	$F_{2,7.18} = 0.90$, $p = 0.447$
RR interval [ms]	131.2 ± 4.36	125.4 ± 3.31	120.3 ± 3.30	$F_{2,16.50} = 1.97$, $p = 0.171$	101.0 ± 5.53	107.9 ± 3.19	111.0 ± 6.28	$F_{2,7.08} = 0.76$, $p = 0.503$
PR interval [ms]	36.4 ± 1.34	34.5 ± 1.32	33.9 ± 1.42	$F_{2,17.27} = 0.92$, $p = 0.417$	29.1 ± 1.98	33.0 ± 2.50	30.3 ± 2.40	$F_{2,9.00} = 0.69$, $p = 0.527$
P duration [ms]	14.6 ± 0.75	12.8 ± 1.04	13.6 ± 1.19	$F_{2,17.52} = 1.09$, $p = 0.358$	13.7 ± 1.42	12.7 ± 1.17	14.6 ± 1.27	$F_{2,8.89} = 0.58$, $p = 0.582$
QRS interval [ms]	10.3 ± 0.31	11.6 ± 0.79	9.91 ± 0.72	$F_{2,16.41} = 1.41$, $p = 0.273$	17.6 ± 1.76	14.5 ± 1.01	17.0 ± 3.24	$F_{2,6.36} = 1.18$, $p = 0.367$
QT interval [ms]	52.3 ± 0.59	51.4 ± 1.19	48.3 ± 1.94	$F_{2,15.82} = 1.87$, $p = 0.186$	60.1 ± 2.89	63.8 ± 1.29	66.1 ± 2.09	$F_{2,7.38} = 1.30$, $p = 0.329$
QTc interval [ms]	45.8 ± 1.09	46.1 ± 1.28	44.1 ± 1.74	$F_{2,17.10} = 0.44$, $p = 0.654$	59.8 ± 1.81	61.5 ± 1.26	62.8 ± 0.95	$F_{2,9.73} = 1.08$, $p = 0.377$
JT interval [ms]	42.0 ± 0.69	39.8 ± 1.52	38.4 ± 2.18	$F_{2,15.78} = 1.73$, $p = 0.209$	42.5 ± 1.40	49.3 ± 1.08	49.0 ± 4.58	$F_{2,6.47} = 6.88$, $p = 0.025$
TpTe interval [ms]	35.6 ± 3.03	33.9 ± 2.71	31.6 ± 3.30	$F_{2,16.79} = 0.40$, $p = 0.678$	25.5 ± 3.04	31.6 ± 2.29	28.0 ± 5.96	$F_{2,6.84} = 1.21$, $p = 0.355$
P amplitude [mV]	0.045 ± 0.004	0.024 ± 0.011	0.057 ± 0.008	$F_{2,17.03} = 2.69$, $p = 0.097$	0.224 ± 0.075	0.122 ± 0.041	0.036 ± 0.010	$F_{2,8.69} = 4.68$, $p = 0.042$
R amplitude [mV]	0.787 ± 0.069	0.648 ± 0.063	0.072 ± 0.100	$F_{2,16.29} = 1.06$, $p = 0.368$	0.248 ± 0.078	0.107 ± 0.035	0.030 ± 0.015	$F_{2,9.21} = 5.06$, $p = 0.033$
S amplitude [mV]	-0.277 ± 0.054	-0.230 ± 0.041	-0.350 ± 0.050	$F_{2,16.16} = 1.67$, $p = 0.219$	-1.887 ± 0.463	-1.130 ± 0.307	-0.517 ± 0.062	$F_{2,8.60} = 5.64$, $p = 0.027$
T amplitude [mV]	0.050 ± 0.027	0.037 ± 0.014	0.028 ± 0.022	$F_{2,14.19} = 0.19$, $p = 0.829$	-0.430 ± 0.064	-0.403 ± 0.106	-0.163 ± 0.026	$F_{2,9.70} = 8.69$, $p = 0.007$

TpTe, Tpeak-to-Tend.

Data are shown as mean±SEM.

Table S9: Gene expression analysis in myocardial tissue 3 days post-MI.

	5-HTT+/+ $n = 7$	5-HTT+/- $n = 9$	5-HTT-/- $n = 9$	Statistics
TGF-β	1.000 ± 0.581	1.428 ± 0.434	3.050 ± 0.246	$F_{2,12.10} = 8.40$, $p = 0.005$
TNF-α	1.000 ± 0.299	1.473 ± 0.312	1.892 ± 0.124	$F_{2,11.53} = 3.96$, $p = 0.049$
IL-6	1.000 ± 0.151	1.333 ± 0.309	1.737 ± 0.255	$F_{2,13.33} = 3.04$, $p = 0.082$
IL-10	1.000 ± 0.487	1.754 ± 0.549	1.961 ± 0.417	$F_{2,14.17} = 1.12$, $p = 0.353$
TH	1.000 ± 0.398	1.905 ± 1.024	0.419 ± 0.077	$F_{2,9.56} = 1.91$, $p = 0.200$
MMP-2	1.000 ± 0.123	1.201 ± 0.150	1.691 ± 0.149	$F_{2,14.65} = 6.21$, $p = 0.011$
MMP-3	1.000 ± 0.100	0.585 ± 0.084	0.899 ± 0.140	$F_{2,14.00} = 5.19$, $p = 0.021$
MMP-13	1.000 ± 0.101	1.293 ± 0.165	0.842 ± 0.064	$F_{2,12.71} = 3.45$, $p = 0.064$
COL-1α1	1.000 ± 0.061	0.963 ± 0.120	0.839 ± 0.104	$F_{2,14.15} = 0.86$, $p = 0.445$
COL-1α2	1.000 ± 0.059	1.182 ± 0.189	0.843 ± 0.122	$F_{2,13.14} = 1.19$, $p = 0.335$
COL-3	1.000 ± 0.097	0.993 ± 0.175	0.992 ± 0.091	$F_{2,14.07} = 0.00$, $p = 0.998$
SPARC	1.000 ± 0.083	0.909 ± 0.118	1.109 ± 0.088	$F_{2,14.50} = 0.94$, $p = 0.413$
ACTA2	1.000 ± 0.181	0.838 ± 0.144	0.887 ± 0.100	$F_{2,12.98} = 0.24$, $p = 0.792$
5-HT2A	1.000 ± 0.082	0.970 ± 0.119	0.851 ± 0.211	$F_{2,14.01} = 1.00$, $p = 0.392$
5-HT2B	1.000 ± 0.259	0.836 ± 0.173	0.679 ± 0.131	$F_{2,12.82} = 0.67$, $p = 0.527$
SERT	1.000 ± 0.366	0.935 ± 0.432	0.000 ± 0.000	$\chi^2_{(2)} = 9.75$, $p = 0.008$

TGF-β, transforming growth factor β; TNF-α, tumor necrosis factor α; IL-6, interleukin 6; IL-10, interleukin 10; TH, tyrosine hydroxylase; MMP-2, matrix metalloproteinase 2; MMP-3, matrix metalloproteinase 3; MMP-13, matrix metalloproteinase 13; Col-1α1, collagen 1α1; Col-1α2, collagen 1α2; Col-3, collagen 3; SPARC, secreted protein acidic and rich in cysteine; ACTA2, alpha smooth muscle actin 2, 5HT2A, serotonin receptor 2A; 5HT2B, serotonin receptor 2B; SERT, serotonin transporter.
Data are shown as mean±SEM.

Table S10: Quantification of neurotransmitter concentrations in myocardial tissue 3 days post-MI.

	5-HTT+/+ n = 8	5-HTT+/- n = 4	5-HTT-/- n = 6	Welch's ANOVA
**Concentration [ng/g]**				
NE	5049 ± 967	3889 ± 773	3690 ± 879	$F_{2,9.40}$ = 0.58, p = 0.577
EPI	78 ± 18	180 ± 79	39 ± 9	$F_{2,6.22}$ = 2.95, p = 0.125
MHPG	105 ± 17	259 ± 98	73 ± 14	$F_{2,6.49}$ = 2.42, p = 0.164
5-HTP	601 ± 15	777 ± 254	699 ± 152	$F_{2,5.06}$ = 0.39, p = 0.694
5-HT	53 ± 10	53 ± 15	39 ± 6	$F_{2,7.05}$ = 0.94, p = 0.433
5-HIAA	77 ± 12	74 ± 12	62 ± 12	$F_{2,8.87}$ = 0.38, p = 0.693
DA	37 ± 5	54 ± 14	23 ± 8	$F_{2,6.64}$ = 2.03, p = 0.205
DOPAC	34 ± 6	53 ± 13	26 ± 7	$F_{2,7.04}$ = 1.59, p = 0.270
HVA	124 ± 16	153 ± 25	139 ± 12	$F_{2,7.37}$ = 0.52, p = 0.612
**Turnover rate**				
MHPG/NE	0.024 ± 0.004	0.072 ± 0.025	0.024 ± 0.006	$F_{2,6.27}$ = 1.64, p = 0.268
MHPG/EPI	1.590 ± 0.235	1.688 ± 0.245	2.116 ± 0.349	$F_{2,8.78}$ = 0.75, p = 0.501
MHPG/(NE+EPI)	0.024 ± 0.004	0.067 ± 0.023	0.024 ± 0.006	$F_{2,6.29}$ = 1.66, p = 0.264
5-HIAA/5-HT	1.860 ± 0.421	1.656 ± 0.365	1.795 ± 0.431	$F_{2,9.26}$ = 0.07, p = 0.936
DOPAC/DA	0.979 ± 0.138	1.042 ± 0.197	1.353 ± 0.290	$F_{2,7.73}$ = 0.63, p = 0.560
HVA/DA	4.617 ± 1.666	3.099 ± 0.532	9.465 ± 2.530	$F_{2,8.63}$ = 3.05, p = 0.099
(DOPAC+HVA)/DA	5.596 ± 1.682	4.142 ± 0.663	10.82 ± 2.730	$F_{2,8.89}$ = 2.79, p = 0.114

NE, norepinephrine; EPI, epinephrine; MHPG, 3-methoxy-4-hydroxyphenylglycol; 5-HTP, 5-hydroxytryptophan; 5-HT, 5-hydroxytryptamine; 5-HIAA, 5-hydroxyindoleacetic acid; DA, dopamine; DOPAC, 3,4-dihydroxyphenylacetic acid; HVA, homovanillic acid.
Data are shown as mean±SEM.

Supplemental Material Figures

Figure S1: Echocardiographic measurements of sham, surviving MI mice with small and large infarction size and non-surviving MI mice at day 1 after surgery.

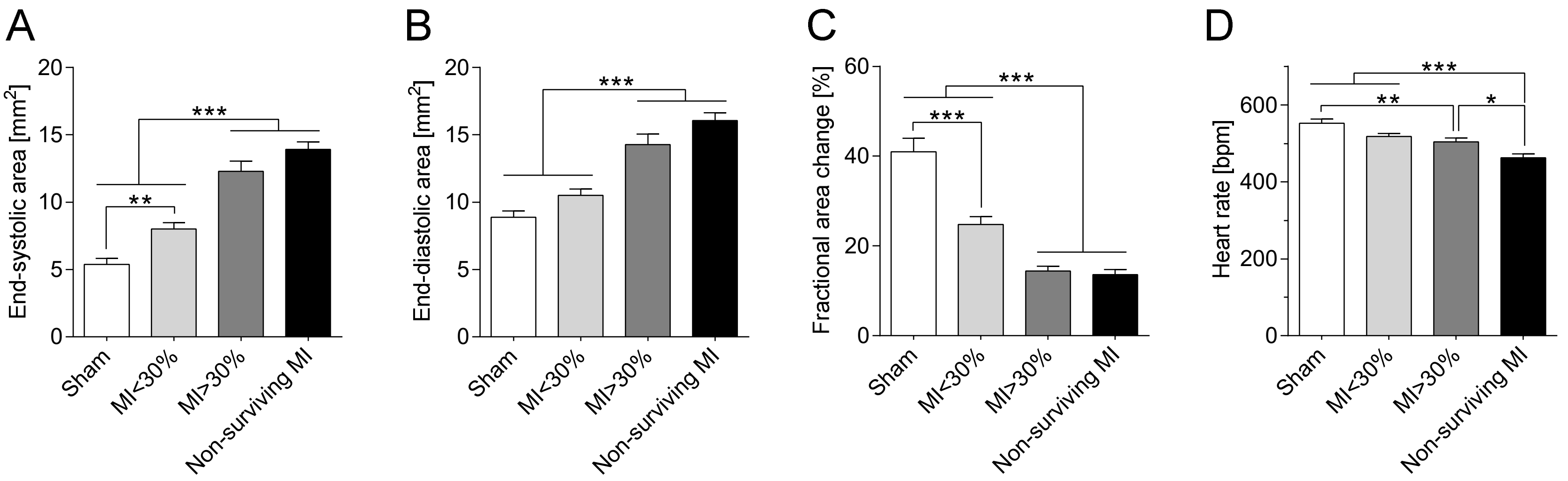

Figure S1: Echocardiographic measurements of sham (*n* = 23), surviving MI mice with small (MI<30%; *n* = 23) and large (MI>30%; *n* = 16) infarct size and non-surviving MI mice (*n* = 28) at day 1 post-op. (A) End-systolic area and (B) end-diastolic area were significantly increased in MI mice relative to sham controls in an infarct size-related manner. (C) Fractional area changes and (D) heart rate were infarct size-dependently decreased in MI mice as compared to sham animals. (A, B) The extent of LV dilatation and (C) the severity of LV dysfunction was comparable among surviving MI>30% and non-surviving MI mice. (D) However, heart rate was significantly reduced in non-surviving vs. MI>30% mice. Data are shown as mean±SEM. $^{*}p < 0.05$, $^{**}p < 0.01$, $^{***}p < 0.001$ (one-way ANOVA followed by Tukey (A, B, D) or Games-Howell (C) post hoc test).

Figure S2: Assessment of body weight change, liquid intake and sucrose preference in sham, surviving MI mice with small and large infarct size and non-surviving MI mice during the first postoperative week.

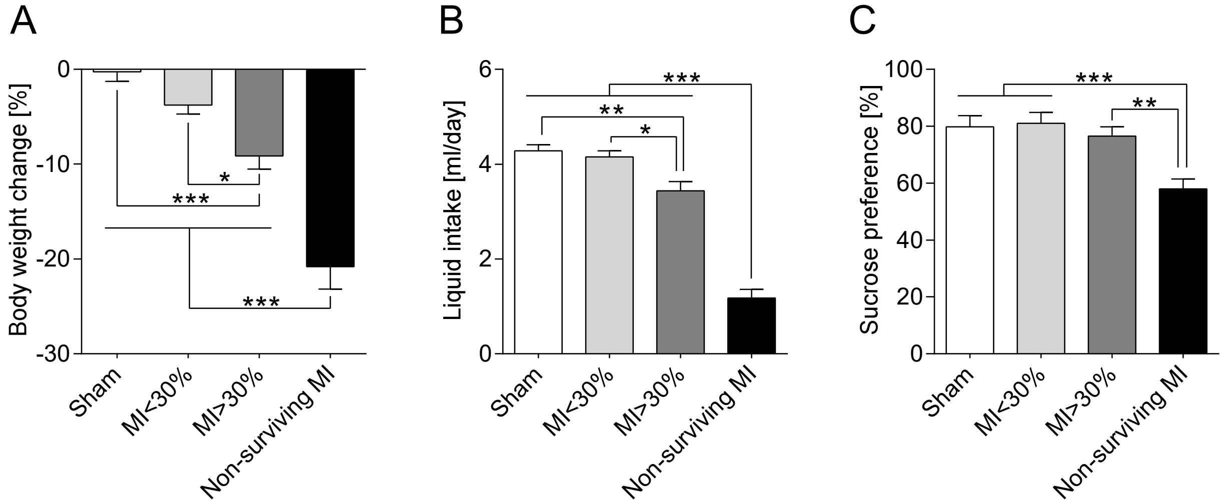

Figure S2: Assessment of body weight change, liquid intake and sucrose preference in sham (n = 23), surviving MI mice with small (MI<30%; n = 23) and large (MI>30%; n = 16) infarct size and non-surviving MI mice (A: n = 9, B-C: n = 18) during the first postoperative week. (A) Body weight and (B) overall fluid consumption were infarct size-dependently decreased in MI mice relative to sham controls (MI>30% < MI<30% < sham). Non-surviving MI mice exhibited the most pronounced body weight loss (A), decrease in liquid consumption (B) and lack of sucrose preference (C) as compared to the other groups, indicating severe sickness behavior due to acute heart failure in these mice. Data are shown as mean±SEM. *$p < 0.05$, **$p < 0.01$, ***$p < 0.001$ (one-way ANOVA followed by Tukey post hoc test).
